# Hidden partnerships in the dark: Cold‐water coral–fish associations in Fiordland, New Zealand

**DOI:** 10.1111/jfb.70330

**Published:** 2026-01-18

**Authors:** Alexander H. Knorrn, Peter Marriott, Kareen Schnabel, André Freiwald

**Affiliations:** ^1^ Senckenberg am Meer, Department of Marine Research Wilhelmshaven Germany; ^2^ Earth Sciences New Zealand Wellington New Zealand; ^3^ Center for Marine Environmental Sciences, University of Bremen Bremen Germany

**Keywords:** fish behaviour, fish ecology, *Lepidoperca tasmanica*, *Madrepora oculata*

## Abstract

The ichthyofauna of the Fiordland ecosystems of southern Aotearoa New Zealand was documented during four remotely operating vehicle (ROV) dives between 100 and 350 m depth. A total of 26 fish species were documented within two fiord basins. An association between cold‐water corals, such as the stony coral *Madrepora oculata* and black coral *Antipathella fiordensis*, and several fish species was observed. In particular, the observed association between *M. oculata* and the wavyline perch (*Lepidoperca tasmanica*) highlighted the use of *M. oculata* colonies not only as sheltering habitats but also as nurseries for this species in Fiordland.

## 1 INTRODUCTION

Reef‐building scleractinian corals provide a foundational source for a wide range of associated fauna (Siqueira et al., 2023). One part of this associated fauna is fish, which act as key components of these scleractinian‐built communities and rely on corals for shelter and food provision (Coker et al., 2014; Pratchett et al., 2008). The dependency of some fishes on corals is also evident from an evolutionary perspective, as reef‐associated fish species are thought to diversify at a significantly faster rate than their non‐reef‐associated counterparts (Alfaro et al., 2007; Cowman & Bellwood, 2011). Therefore, it is not surprising that coral–fish associations are found not only in euphotic shallow‐water environments but also in aphotic habitats built by azooxanthellate cold‐water corals.

During the 309th cruise of the German research vessel SONNE, several deep‐water coral habitats around the South Island of Aotearoa New Zealand were investigated. These cold‐water coral habitats were observed using the MARUM remotely operating vehicle (ROV) *Squid 2000* manufactured by SAAB Seaeye. The ROV was equipped with a SULIS Z70 camera system, which provides 4 K UHD video resolution at 30 fps, as well as an HD Lookdown prototype camera manufactured by MARUM. Individual 14‐megapixel pictures were taken using an Imenco TIGERSHARK camera. Lighting was provided by two SAAB LEDs mounted parallel to the operating cameras, and two Imenco Dusky Shark line lasers were installed as a 10‐cm reference during the filming process. A ship‐mounted Sonardyne RANGER II USBL system was used to position the ROV during its operation. All fish sightings were recorded and identified to the species level using relevant ichthyological identification literature, such as Roberts et al. (2015), McMillan et al. (2019) and Francis (2024).

Several dives during cruise SO309 were conducted within the Fiordland (Te Moana o Atawhenua) Marine Area, which was formed during the Pleistocene glaciation period and reached a glacial maximum in Aotearoa New Zealand between 26,000 and 18,000 years ago (Cotton, 1956; Pillans et al., 1992; Trewick & Wallis, 2001; Wing & Jack, 2014). The area was designated as a UNESCO World Heritage Site in 1973 (Bonehill, 2006). Like other fjords in temperate and subpolar areas around the world, Fiordland shows a remarkably different fauna to offshore regions in its close proximity (Brattegard et al., 2010). Today, the majority of research conducted in Aotearoa's New Zealand's fjords covers the benthic communities of shallow subtidal and intertidal habitats (Bell et al., 2024; Brewin et al., 2008; Kelly et al., 2021). Deeper regions of the fiord systems still need to be investigated in more detail.

**FIGURE 1:**
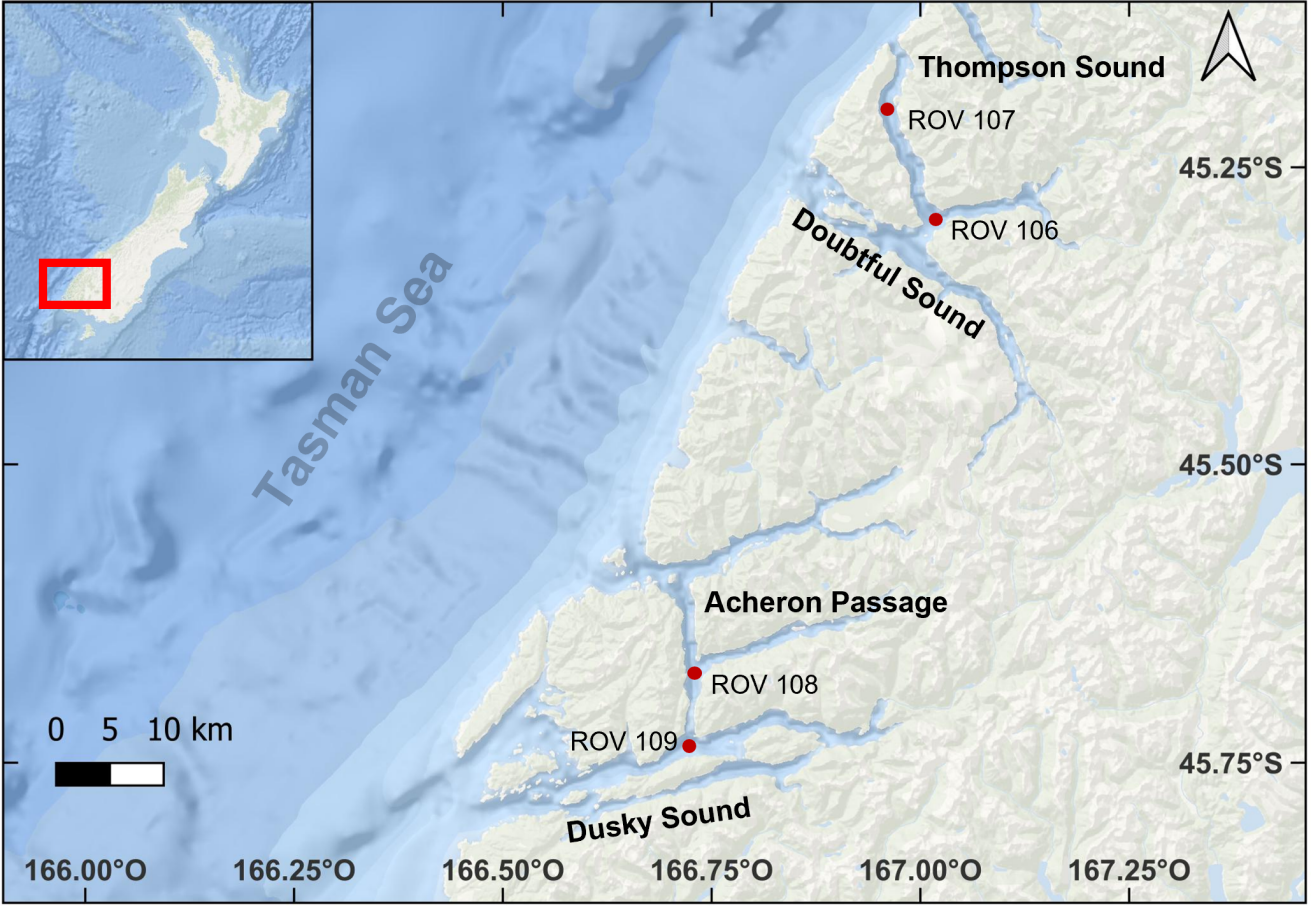
Map depicting the research area referred to in this paper. The red frame in the inset map indicates the location of the Fiordland research area in southern Aotearoa New Zealand. Red dots indicate the sites of the MARUM ROV *Squid* dives. Basemap from ESRI Ocean, 2025.

Four different ROV dives were conducted in the Fiordland area during SO309; see Freiwald et al. (2025) for more details:

Dive 106 (GeoB26344) was conducted in the central Thompson Sound area and began at a depth of 350 m (Figure 1). The soft‐bottom habitat was mainly bioturbated by burrowing fauna, possibly by gebiid crustaceans (mud shrimp). An abrupt transition to steep rock walls occurred at a depth of 328 m. Dense populations of pseudocolonial dendrophylliid cold‐water corals appeared immediately after the transition to hard substrates. At a depth of 315 m, the rock walls formed vertical sections inhabited by the ostreid bivalve, *Pulvinites exempla*.

Dive 107 (GeoB26349) was deployed on the southern slope of a glacial sill, known as Pendulo Reach, at depths ranging between 245 and 112 m (Figure 1). This sill has been designated as a protected area by the Department of Conservation since 2011 due to its vulnerable coral populations (stylasterids, octocorals, antipatharians and scleractinians) (FMG, 2024). During this dive at depths between 134 and 130 m, a 60‐cm‐tall living *Madrepora oculata* colony was observed in which several fish species, including the wavyline perch (*Lepidoperca tasmanica*), were sheltering. In the shallowest part of the dive (130–112 m), a 1‐m‐tall endemic black coral (*Antipathella fiordensis*) was observed, and its associated fauna documented.

Dive 108 (GeoB26356) was conducted around an east‐facing slope in the central area of the Acheron Passage and covered a depth range of 231–100 m. Occasional 10–30‐cm‐high colonies of *M. oculata* were found, along with numerous stylasterids and scattered *Eguchipsammia* corals. A sediment‐covered terrace was observed at a depth of 185 m, and another cliff was observed at a depth of 105 m. With decreasing depth, the abundance of acanthogorgiid corals increased significantly, and additional *A. fiordensis* colonies were seen at approximately 165 m depth.

Dive 109 (GeoB26361) was conducted over a rocky outcrop at the eastern entrance to Bowen Channel in Dusky Sound and covered a bathymetric range from 289 to 102 m. Several shell‐rich aprons were documented, consisting primarily of fallen shells of *Acesta* sp., *P. exempla*, dead *M. oculata* colonies and some live *Eguchipsammia* corals mixed with cobbles. Occasionally, highly bioluminescent solitary *Caryophyllia* corals were observed, and an area dotted with small, stalked hexactinellid glass sponges *Stylocordyla* sp. was documented at a depth of 142 m.

A total of 26 different fish species were observed during the four dives in the Fiordland area. A detailed list of all observed fish species is presented in Table 1.

**TABLE 1:** Observed fish species of Fiordland during four different dives of the MARUM ROV *Squid* conducted during the R.V. *SONNE Cruise SO309* in the Fiordland National Park.

Species	Common names	106	107	108	109
*Eptatretus cirrhatus*	Common hagfish	X			X
*Mustelus lenticulatus*	Rig				X
*Squalus acanthias*	spiny dogfish		X		
*Dipturus innominatus*	Smooth skate	X			
*Argentina elongata*	Silverside		X		
*Maurolicus australis*	Pearlside	X			X
*Trypterigidae indet*	Triplefin		X		
*Tripterygion oculus*	Ocelate triplefin		X		
*Coelorinchus aspercephalus*	Obliquebanded rattail	X			X
*Coelorinchus biclinozonalis*	Twosaddle rattail		X		
*Coelorinchus bollonsi*	Bollons’ rattail				
*Coelorinchus fasciatus*	Banded rattail	X	X	X	
*Coelorinchus oliverianus*	Oliver's rattail	X			
*Coelorinchus parvifasciatus*	Smallbanded rattail	X			
*Paratrachichthys trailli*	Common roughy			X	
*Capromimus abbreviatus*	Capro dory	X			
*Helicolenus* cf. *barathri*	Bigeye sea perch	X	X	X	X
*Caesioperca lepidoptera*	Butterfly perch		X	X	
*Lepidoperca aurantia*	Orange perch		X	X	X
*Lepidoperca tasmanica*	Wavyline perch				X
*Nemadactylus macropterus*	Tarakihi		X		
*Notolabrus cinctus*	Girdled wrasse		X	X	X
*Pseudolabrus miles*	Scarlett wrasse		X	X	X
*Parapercis colias*	Blue cod		X		
*Hemerocoetes monopterygius*	Opalfish		X		
*Arnoglossus scapha*	Witch		X		

**FIGURE 2:**
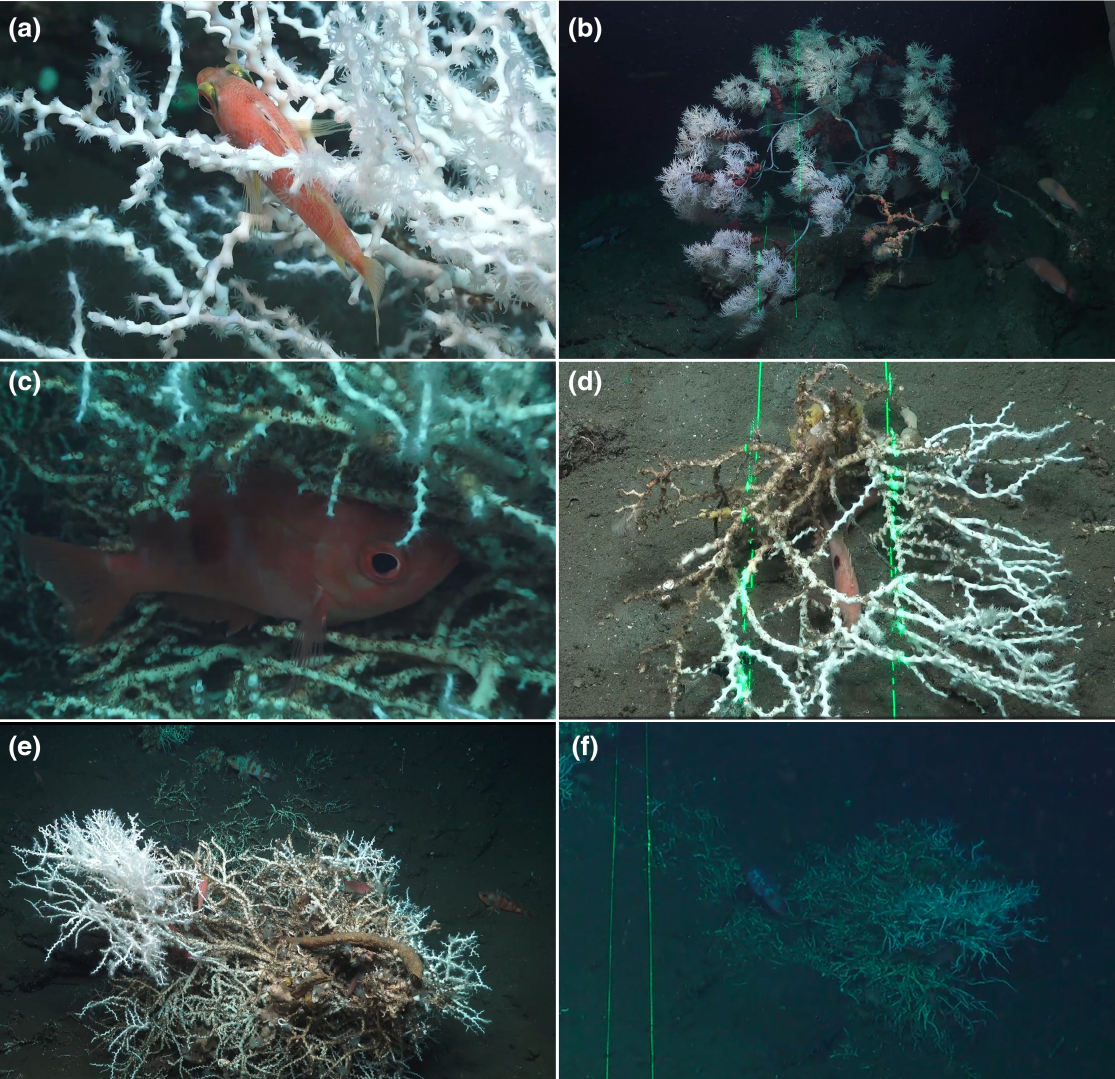
Fish–coral associations documented during ROV dive 107 in Doubtful Sound. (a) *Lepidoperca tasmanica* sheltering between *Madrepora oculata* branches. (b) A large *Antipathella fiordensis* inhabited by brittle stars (*Astrobrachion constrictum*), *Notolabrus cinctus* and a *Pseudolabrus miles*. (c) *Lepidoperca aurantia* hiding between the *M. oculata* branches. (d) *Caesioperca lepidoptera* hiding between the branches of a smaller *M. oculata* colony. (e) *M. oculata* colony inhabited by *L. tasmanica* and two *Helicolenus* cf. *barathri* resting on the seafloor behind the colony. (f) *Parapercis colias* going to shelter underneath a *M. oculata* colony.

**FIGURE 3:**
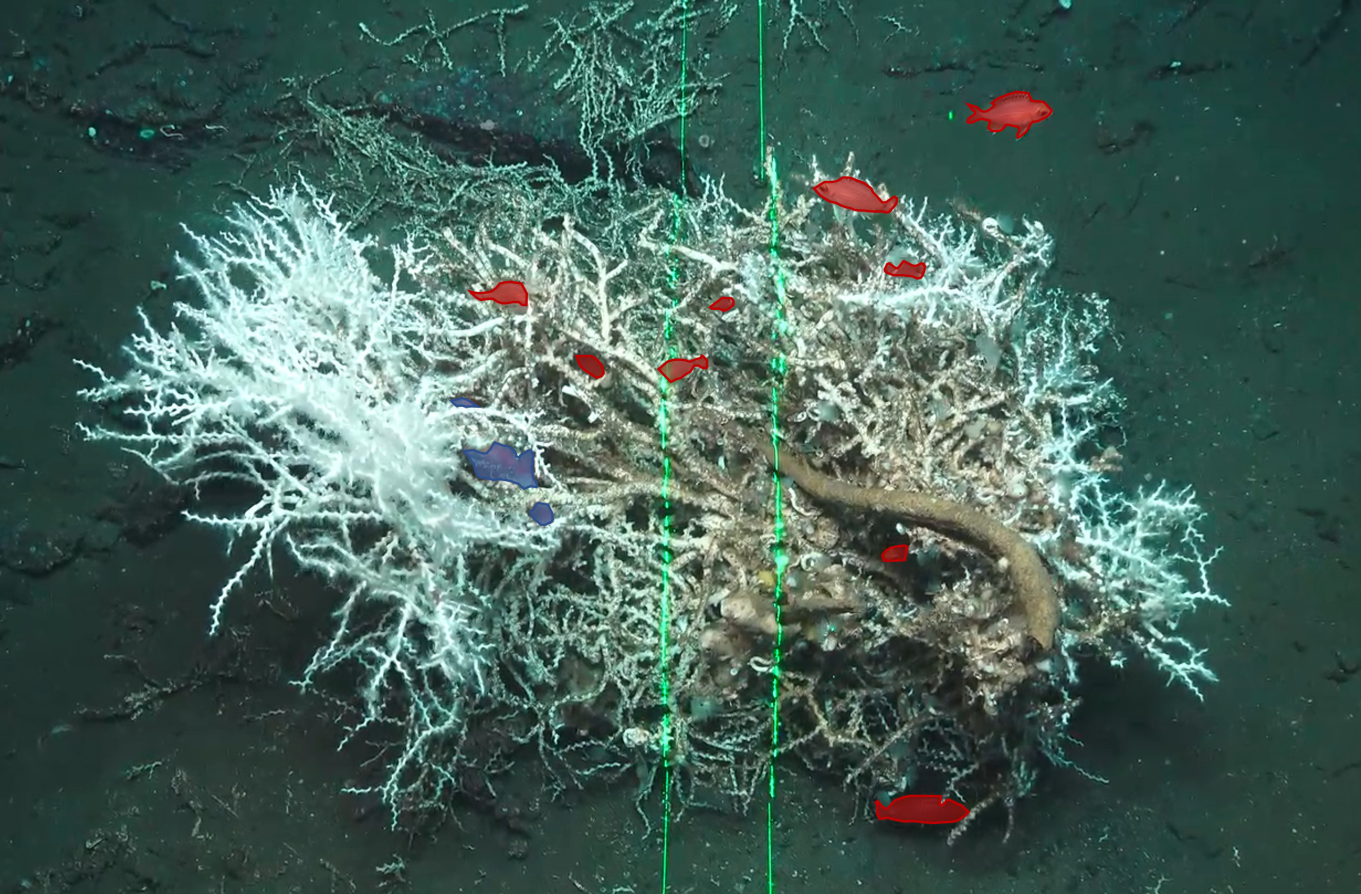
A large *Madrepora oculata* colony from Doubtful Sound at a depth of 134–130 m. Reddish silhouettes represent visible *Lepidoperca tasmanica* individuals (*N* = 9) in close proximity to the coral colony. Blue silhouette represents a larger *Caesioperca aurantia* individual hiding within the coral framework.

In particular, Dive 107 revealed associations between deep‐water corals and different fish species. For instance, several fish species were associated with *M. oculata*. One orange perch individual (*Lepidoperca aurantia*) was observed hiding under a dense framework of *M. oculata* (Figure 2c). A smaller butterfly perch individual (*Caesioperca lepidoptera*) inhabited a smaller *M. oculata* framework, approximately 5 m apart from the larger colony (Figure 2d). *L. aurantia* was identified by the prominent dark pinkish blotch below the lateral line near the tip of the pectoral fin and the steeply curved prominent lateral‐line scales. *C. lepidoptera* was identified by its brownish‐red to orange colour and conspicuous dark brownish blotch on each flank of its body, as well as through several small dark spots along the dorsal and lateral sides of its body. Several wavyline perches (*L. tasmanica*) were also detected living within the larger *M. oculata* colony (Figures 2a and 3). *L. tasmanica* was identified by its orange body with pale pink scales above the lateral line. The scales of the lateral line and below were silvery white with pink margins and produced a honeycomb pattern. The large coral colony appeared to serve as a refuge for at least nine *L. tasmanica* individuals. Several smaller, presumably subadult individuals also appeared to use this colony as a nursery or shelter. This observation shows a very clear association between *L. tasmanica* and *M. oculata* and confirms the assumption of Roberts (1989) that *L. tasmanica* could be closely associated with corals. Additionally, a blue cod (*Parapercis colias*) was observed as the ROV slowly approached the *M. oculata* colony (Figure 2f). It was identified by its elongated body with a blunt rounded head. The body was coloured rusty brown to light grey with a mottled pattern, which indicated that the observed specimen was a female. The specimen remained hidden underneath the *M. oculata* framework for the remainder of the video recording. Another species observed in close proximity to the large *M. oculata* colony was the bigeye seaperch (*Helicolenus* cf. *barathri*), which was resting on the seafloor 1–2 m apart from the investigated colony (Figure 2e). It was identified by its seaperch‐like body with prominent opercular spines and strongly pronounced dorsal spines. Additionally, the Y‐shaped bands and several diffuse large lines merge at the anterior dorsal parts of its body. However, the closely related jock stewart (*Helicolenus percoides*) can easily be misidentified as *H. barathri*, and only a physical examination that compares the pectoral‐fin rays allows species identification with high confidence. More species observed in association with *A. fiordensis* were the gridled wrasse (*Notolabrus cinctus*) and scarlet wrasse (*Pseudolabrus miles*), which were observed swimming around the black coral at a depth of 130 to 112 m (Figure 2b). *N. cinctus* was identified by its wrasse‐like body and labriform swimming style, which uses the pectoral fins for propulsion and the caudal fin for manoeuvring. Its pale grey to greenish body colouration and a prominent transverse dark black band along the body, which was less pronounced in juvenile individuals, allow a clear identification. *P. miles* is identified by its reddish body and distinctive broad black bar at the base of the caudal fin and its labriform swimming style. *A. fiordensis* was also used by *L. tasmanica* individuals as shelter between the different branches of the coral.

This study not only provides an insight into the ichthyofaunal composition of the deeper Fiordland ecosystems but also highlights previously undocumented coral–fish associations. The number of *L. tasmanica* individuals (*N* = 9), including several juveniles, associated with one large *M. oculata* colony (see Figure 3), highlights the importance of cold‐water corals as a refuge and nursery ground within the Fiordland ecosystem. Additionally, the fish–coral associations observed were all shelter‐type interactions; that is, fish used the three‐dimensional matrix structure of the coral as a refuge to hide or reduce predation potential while at rest. In most of the interactions, the fish were already calmly resting within the coral matrix when first observed. In the case of one blue cod *P. colias* observation, the approaching ROV and its bright lights spooked the fish causing it to flee to a refuge underneath a *M. oculata* colony.

## AUTHOR CONTRIBUTIONS

A. H. K.: Conceptualisation, data collection, writing – original draft, writing – review and editing. P. M.; K. S.: Data collection, writing – review and editing. A. F.: Conceptualisation, data collection, writing–review, project administration, funding acquisition.

## FUNDING INFORMATION

This study was funded by the Ministry of Research, Technology and Space (BMFTR) project CoralNewZ – Cold‐Water Coral Biology and Geology off New Zealand. This project includes ship time on R.V. *SONNE* and *MARUM* ROV *Squid* operations through the grants 03G0309A and 03G0309B.

## CONFLICT OF INTEREST STATEMENT

None of the authors have a conflict of interest to disclose.

## Data Availability

The data that support the findings of this study are available from the corresponding author upon reasonable request.
